# Pathologic properties of SOD3 variant R213G in the cardiovascular system through the altered neutrophils function

**DOI:** 10.1371/journal.pone.0227449

**Published:** 2020-01-31

**Authors:** Myung-Ja Kwon, Kyo-Young Lee, Won-Gug Ham, Lee-Jung Tak, Gaurav Agrahari, Tae-Yoon Kim

**Affiliations:** 1 Department of Dermatology, Catholic Research Institute of Medical Science, College of Medicine, The Catholic University of Korea, Banpo-Dong, Seocho-gu, Seoul, Republic of Korea; 2 Department of Hospital Pathology, College of Medicine, The Catholic University of Korea, 222 Banpo-Dong, Seocho-gu, Seoul, Republic of Korea; Medical College of Georgia at Augusta University, UNITED STATES

## Abstract

The SOD3 variant, SOD3_R213G_, results from substitution of arginine to glycine at amino acid 213 (R213G) in its heparin binding domain (HBD) and is a common genetic variant, reported to be associated with ischemic heart disease. However, little is understood about the role of SOD3_R213G_ in innate immune function, and how it leads to dysfunction of the cardiovascular system. We observed pathologic changes in SOD3_R213G_ transgenic (Tg) mice, including cystic medial degeneration of the aorta, heart inflammation, and increased circulating and organ infiltrating neutrophils. Interestingly, SOD3_R213G_ altered the profile of SOD3 interacting proteins in neutrophils in response to G-CSF. Unexpectedly, we found that G-CSF mediated tyrosine phosphatase, SH-PTP1 was down-regulated in the neutrophils of SOD3_R213G_ overexpressing mice. These effects were recovered by reconstitution with Wt SOD3 expressing bone marrow cells. Overall, our study reveals that SOD3_R213G_ plays a crucial role in the function of the cardiovascular system by controlling innate immune response and signaling. These results suggest that reconstitution with SOD3 expressing bone marrow cells may be a therapeutic strategy to treat SOD3_R213G_ mediated diseases.

## Introduction

Superoxide dismutase 3 (SOD3) is a member of the SOD family that scavenges superoxide and ROS produced by cells and tissues during inflammation [1]. Specifically, SOD3 is a glycoprotein with a heparin-binding domain (HBD) and is distributed throughout the extracellular matrix (ECM) of many tissues, including blood vessels and heart [2–6]. Binding of the HBD to heparan sulfate proteoglycans on cell surfaces and ECM is critical for the function of SOD3 [4], protecting these organs against oxidative stress [4, 7, 8]. We previously reported that SOD3 acts as a signal regulator by modulating innate and adaptive immune responses to ameliorate skin diseases and airway inflammation in mice [9, 10]. Considering that the ECM is essential for regulating intercellular communication [11], SOD3 may play a critical role for maintaining proper cellular function.

SOD3 variant R213G (SOD3_R213G_), the substitution of arginine to glycine at amino acid 213 in the HBD, is a common human gene variant [12, 13] and is known to be associated with many diseases, including ischemic heart disease [13] and vascular impairment [4]. Individuals who carry SOD3_R213G_ exhibit increased plasma concentrations of SOD3 [14, 15], and have increased risk of ischemic heart disease [13]. Furthermore, SOD3_R213G_ is associated with increased triglyceride levels and body weight [12]. Cohort studies showed that diabetic patients who carry SOD3_R213G_ have higher mortality rates, including significantly higher death rates from ischemic heart disease and cerebrovascular disease than non-carriers [16]. In addition, SOD3 gene transfer reduces arterial pressure and improves vascular function (29). However, little is known about the role of SOD3_R213G_ in innate immune function, which causes dysfunction of the cardiovascular system.

Neutrophils are released from the bone marrow (BM) and maintain homeostatic levels in the blood, in respond to infection. Granulocyte-colony stimulating factor (G-CSF) is a potent stimulus for releasing neutrophils from the BM during infection [17]. Binding of G-CSF to its receptor (G-CSFR) activates a number of signaling cascades, which controls granulopoiesis, proliferation, and trafficking of neutrophils [18, 19]. During activation, neutrophils clear pathogens by generating large amounts of ROS through a respiratory burst process [20]. However, the accumulated intracellular ROS damages healthy cells and organs [21]. In addition, activated neutrophils secrete extracellular remodeling substances, such as elastase, cathepsin G, and pro-inflammatory cytokines, which further accelerate tissue damage [22, 23]. Considering that SOD3 is located in the ECM, is highly expressed in the aorta and heart, and attenuates activation of neutrophils and inflammation [24], SOD3 may act as a front-line controller for immune response against microbial invasion.

In this study, we investigated the pathologic properties of SOD3_R213G_ in the cardiovascular system using a transgenic mouse model. We report that SOD3_R213G_ leads to cardiovascular dysfunction with failure maintaining immune response, increasing proliferation and trafficking of neutrophils in the cardiovascular system. Furthermore, SOD3_R213G_ overexpressing neutrophils exhibit altered signaling, in response to G-CSF. However, these effects were recovered by transplantation with Wt SOD3 expressing BM cells. Taken together, these results suggest that arginine at amino acid 213 in the HBD of SOD3 is critical for the function of SOD3.

## Materials and methods

### Animals

All mice, including C57BL/6, SOD3 Tg, and SOD3_R213G_ Tg mice, were used as previously described [10, 25] and cared for in semi-specific pathogen-free conditions. Animal experiments were performed in accordance with established institutional guidance that was approved by the Research Animal Care Committee of Catholic University (Seoul, Korea). The animals were sacrificed by cervical dislocation.

### Quantitative real time-PCR

To perform qRT-PCR, the following mouse primers were used. Neutrophil secreting elastase, 5′-CTCTGGCTGCCATGCTACT, 3′-GTTCACACAGTGGGCTGCT; cathepsin G, 5′CGGCAGCAACT-GACTAAGC, 3′-CAAGCACTCAGCCCTTCTG; TERT, 5′-GTTCCTGTTCTGGCTGATG, 3′-CTTGTG-ACAGCTCCCGTAG; actin, 5’ AGCTGTGGACAAAGCCAACT, 3’-TTGGGCTCTCTCAGTTC-CCAC. Primers used for TNFα, IL-6, IL-1β, MCP-1, MIP1α, MIP1β, and CCR2 were described previously [10, 25]. All qRT-PCR data were normalized against mRNA levels of the actin gene.

### Tissue histology and immunofluorescence staining

The tissues, including aorta and heart, were isolated and fixed in 4% paraformaldehyde in PBS and embedded in paraffin according to general histochemical procedure as previously described [9]. H&E staining, Masson’s staining, and Van Gieson’s staining were performed according the manufacturer’s instructions (Sigma, St Louis, MO). Inflammation, organ degeneration, and flattened and broken elastic fibers were assessed based on pathology criteria. To examine neutrophils infiltration, immunofluorescence staining was performed as previously described [9]. Fluorescence conjugated antibodies against CD11b (BioLegend, San Diego, CA), NIMP-R14 (Abcam, Cambridge, UK), and DAPI for nuclear staining (Vector Lab, Burlingame, CA) were diluted 1:100 and incubated for 1 hour at room temperature. The images were taken by confocal microscopy (Carl Zeiss, Thornwood, NY).

### Blood differential staining

Blood was withdrawed from Wt or SOD3_R213G_ mice. Fifty microliters of blood were subjected to cytospin at 45 rpm for 5 min, followed by Diff-Quick Staining (Sysmex Corporation). Inflammatory cells, including neutrophils, macrophages, or lymphocytes, were classified as previous described [10].

### Cell surface and intracellular staining

APC-conjugated anti-mouse Ly-6G (Gr1, 1A8-Ly6g), PE-conjugated anti-mouse CD11b (M1/70), TNFα (MP6-XT22), and FITC–conjugated anti-mouse IL-6 (MP5-20F3) were purchased from eBioscience (San Diego, CA). PI and FITC-conjugated Annexin V were obtained from BioLegend (San Diego, CA). Cell surface, Gr1, CD11b, Annexin V, and PI were measured by flow cytometry (FACS Calibur, BD Bioscience, San Jose, CA). The content of Gr1+ BM mature neutrophils, and Gr1+ CD11b+ neutrophils of blood and spleen were assessed by representative flow cytometric analysis. The numbers represent the percentage of cells within the gates. At least 10^5^ total cells were acquired by gating on size versus granularity, followed by exclusion of dead cells, and finally detection of markers described in plots. Cytokines TNFα and IL-6 in the spleen were assessed by flow cytometry after intracellular staining with permeabilizing reagent (BD Cytofix/Cytoperm). The data was analyzed with Cell Quest software (Becton Dickinson, Mountain View, CA) as previously described [9].

### Proliferation and apoptosis of BM neutrophils

BM neutrophils were isolated and purified using antibody-magnetic bead depletion with the lineage cell depletion kit (Miltenyi Biotec, Bergisch Gladbach, Germany), and the proliferation was assessed as previously described [25]. The morphology of neutrophils was examined by light microscope. Apoptotic cells were assessed by staining with Annexin V and Propidium Iodide (PI). In some experiments, BM cells were used.

### Neutrophil chemotaxis assay

Transwell plates of 3 μm pore size (Corning Costar, Cambridge, MA) were loaded with 600 μl of medium in the presence of fMLP (100 nM) in the lower chamber. Analyses were run in triplicate and migrated cells in the lower chamber were counted as previously described [25].

### Immunoblot and immunoprecipitation

Antibodies for SH-PTP1 and β-actin were obtained from Santa Cruz Biotechnology (Santa Cruz, CA), antibody for G-CSFR was obtained from Sigma (St. Louis, MO), and human SOD3 antibody was obtained from Abcam (Cambridge, MA). Immunoblot was performed as previously described [25]. Immunoprecipitation was performed using SOD3 antibody (Upstate Biotechnology, Lake Placid, NY) as previously described [10].

### Measurement of ROS content and SOD activity

ROS generation of neutrophils was measured by staining with H_2_DCFH-DA, 2’, 7’- dichlorofluorescin-diacetate (5 μM), followed by FACS analysis as previously described [25]. Tissue or blood SOD activity was measured using a SOD assay kit (Dojindo lab, Kumamoto, Japan).

### BM reconstitution

Seventeen week old SOD3_R213G_ Tg mice were 900 rad γ-irradiated with Cs137 using Gamma cell 3000 Elan (MDS Nordion Co. Inc, Ottawa, Canada). BM stem cells were isolated from same age β-actin promoter driven SOD3 Tg mice as previously described [9]. Twelve hours later, the mice were intravenously injected with BM stem cells (2 ×10^6^ cells). Mice were maintained on antibiotic water one day prior and one week after transplantation. Two months later, the mice were sacrificed for further analysis.

### Mass spectrometry analysis

The neutrophils isolated from Wt or SOD3_R213G_ Tg mice were treated with G-CSF (100 ng/mL). The cells were lysed for immunoprecipitation with an antibody against SOD3. The immunoprecipitation product was subjected to SDS-PAGE on 4–20% gradient gel, followed by Coomassie staining (S1 Fig). Bands around 35–47 and 75–95 kDa were removed from the gel for MS analysis as previously described [10]. The quantitative data was determined using Scaffold proteomics software (Proteome Software, Inc.). Identified proteins were obtained by 90% cut off range and unknown proteins were excluded.

### Statistical analysis

The data was represented as the mean and standard error (SE) and analyzed by ANOVA at *p*<0.05 and Schiffe’s post hoc test, or *t*-test (*p*<0.001). Differences with *p* value below the stated thresholds were regarded as statistically significant.

## Results

### SOD3_R213G_ Tg mice exhibit vascular pathologic changes including cystic medial degeneration and heart inflammation

**Fig 1:**
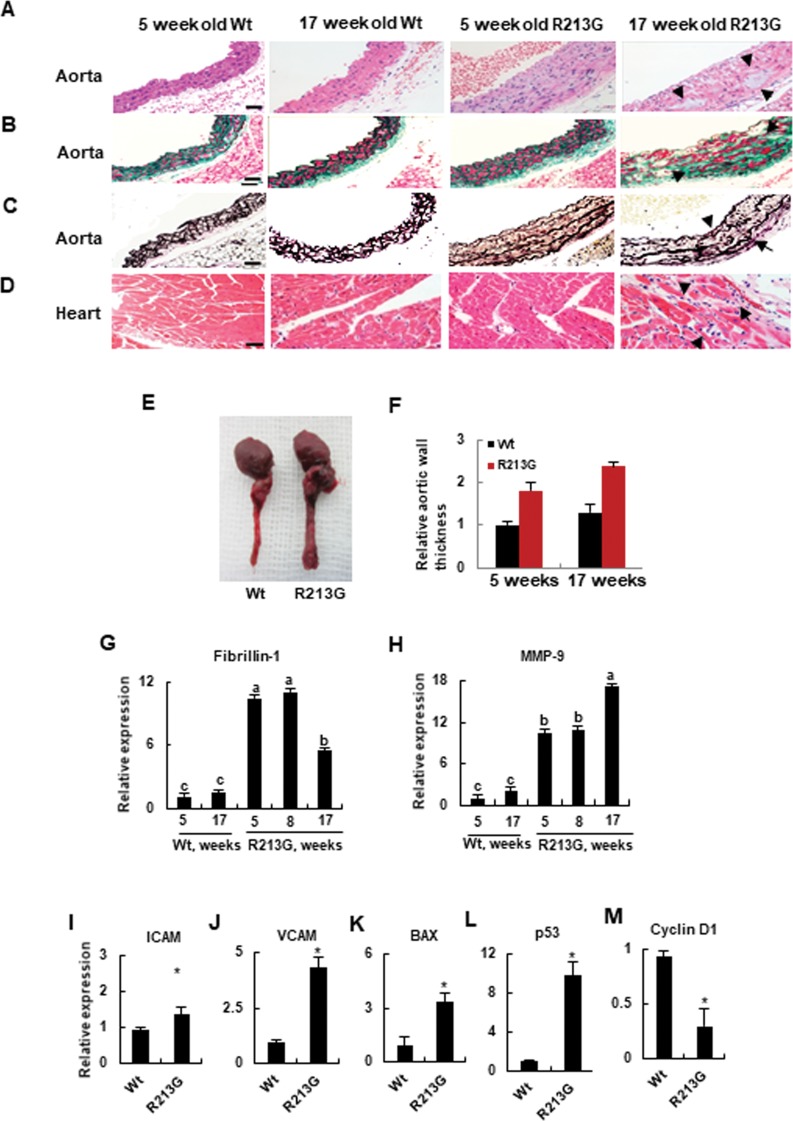
Aortic degeneration and heart inflammation are presented in SOD3_R213G_ mice. **A-D.** Aortic degeneration and heart inflammation in SOD3_R213G_ mice. Abdominal aorta (A-C) and heart (D) of 17 week old SOD3_R213G_ and Wt mice were isolated, along with those of 5 week old SOD3_R213G_ and Wt mice. H & E (A and D), Masson’s trichrome (B), and Van Geison’s staining (C) were performed. Scale bar, 50 μM. Arrow indicates atrophic myocytes (A, 4th panel), fibroblastic proliferation and hydropic degeneration (B, 4th panel), flattened and broken elastic fibers in the aorta (C, 4th panel), and infiltrated inflammatory cells in the heart (D, 4th panel) in the 17 week SOD3_R213G_ mice. **E**. Image of the aorta. **F**. Relative aorta wall thickness. Image of the aorta (E) was taken with a digital camera and relative aorta wall thickness (F) was measured. All images are representative of at least of three independent experiments. **G-M**. Gene expression in the aorta. Fibrillin-1 (G), MMP-9 (H), ICAM (I), VCAM (J), BAX (K), p53 (L), and Cyclin D1 (M). The gene expression was measured by qRT-PCR. All qRT-PCR data were normalized against the mRNA level of the actin gene. The data represent the mean and SE of at least three independent experiments. Statistical analysis was performed by using ANOVA at *p*<0.05, or *t*- test at *p*<0.001, followed by Scheffe’s post hoc test.

In 17 week old SOD3_R213G_ Tg mice, numerous pathological changes in the cardiovascular system were observed. As shown in Fig 1, cystic medial degeneration, including severe atrophic changes of aortic myocytes (Fig 1A, 4th panel), fibroblastic proliferation including increased interstitial collagen and separated myocytes (Fig 1B, 4th panel), hydropic degeneration (Fig 1B, 4th panel), and flattened and broken elastic fibers (Fig 1C, 4th panel) were observed in 17 week old SOD3_R213G_ Tg mice, indicating that the mice were predisposed to aortic dissection or aneurism. In addition, heart inflammation was observed in 17 week old SOD3_R213G_ Tg mice (Fig 1D, 4th panel). Furthermore, 17 week old SOD3_R213G_ Tg mice had bigger hearts and thicker arterial vessels than Wt mice (Fig 1E and 1F). However, these phenotypes were not observed in 5 week old SOD3_R213G_ Tg mice or Wt mice, indicating that the pathology is a progressive development after environmental exposure (Fig 1A–1D, right panel). These histological analyses were further confirmed by assessing the expression of corresponding genes in the aorta: fibrosis and elastin break related genes, fibrullin-1 and MMP9 (Fig 1G and 1H); molecules for recruiting pro-inflammatory substances, ICAM and VCAM (Fig 1I and 1J); apoptosis related genes, Bax (Fig 1K), p53 (Fig 1L), and Cyclin D1 (Fig 1M). Therefore, these results suggest that 17 week old SOD3_R213G_ Tg mice exhibit cardiovascular pathologic changes.

### Neutrophils highly infiltrate in the aorta and heart of SOD3_R213G_ Tg mice

**Fig 2:**
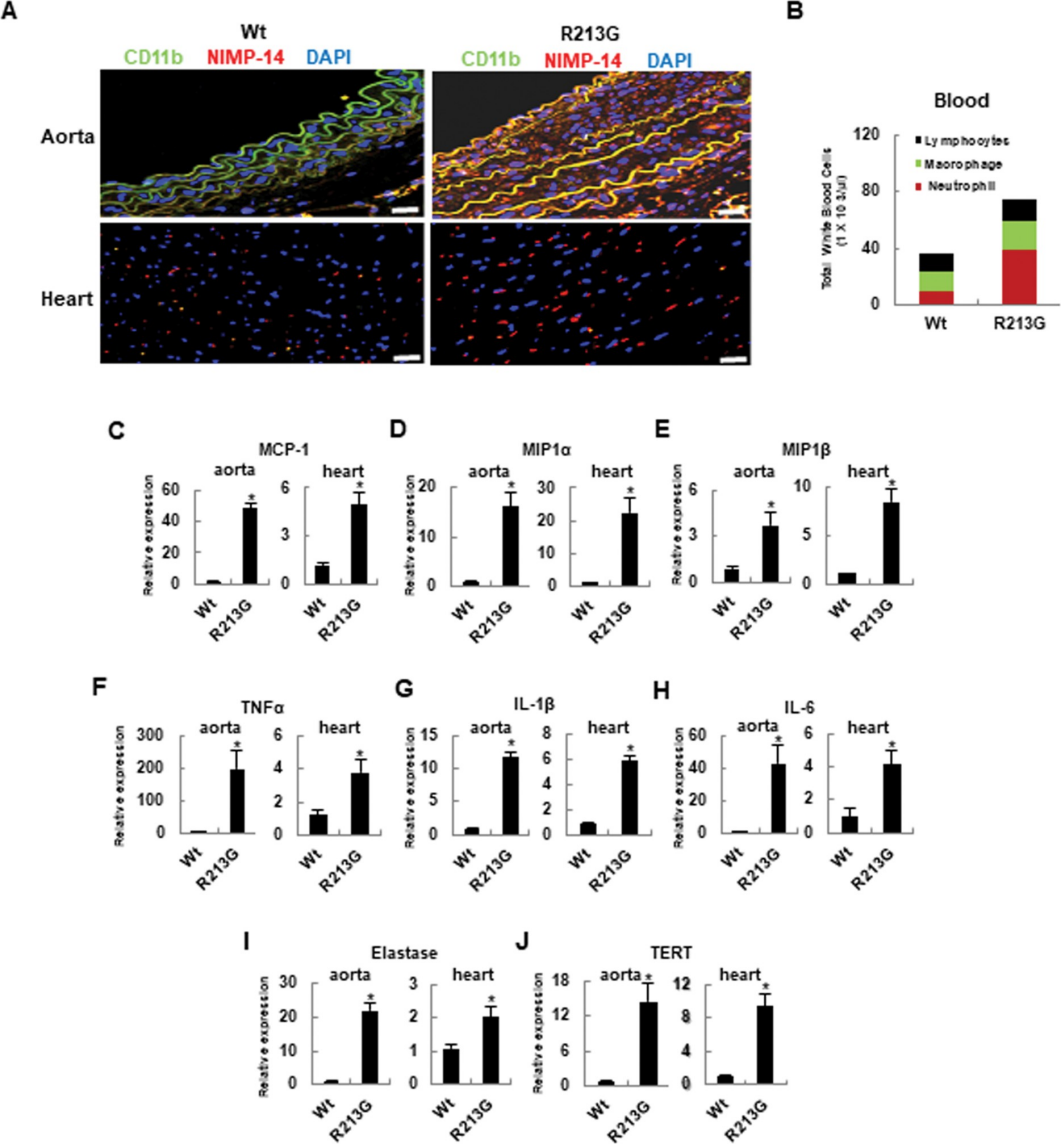
Neutrophils are highly infiltrated in the aorta and heart of SOD3_R213G_ mice. **A**. Neutrophils infiltration in the aorta and heart of SOD3_R213G_ mice. Abdominal aorta (upper panel) and heart (lower panel) of 17 week old SOD3_R213G_ or Wt mice were isolated, and immunofluorescence staining and confocal analysis were performed as described in Materials and Methods. NIMP-R14: marker for peripheral neutrophils; CD11b: marker for monocytes, neutrophils, granulocytes, and macrophages. Scale bar, 100 μM. **B**. Profiles of blood cells. Blood was withdrawn from Wt or SOD3_R213G_ mice, followed by Diff-Quick Staining. Inflammatory cells, including neutrophils, macrophages, or lymphocytes, were classified as described in Materials and Methods. **C-E**. Chemo-attractant expressions **F-H**. Pro-inflammatory cytokine expressions, **I** and **J**. Elastase and TERT expressions in the aorta and heart. Chemoattractants MCP-1 (C), MIP1α (D), MIP1β (E), and proinflammatory cytokines TNFα (F), IL-1β (G), IL-6 (H), and elastase (I), and TERT (J) expression in the aorta and heart of 17 week old SOD3_R213G_ or Wt mice was assessed by qRT PCR. Immunofluorescence data are representative of at least three independent experiments. All qRT-PCR data were analyzed as Fig 1I–1M. Statistical analysis was performed by *t*- test (*p<0.001).

As shown in the result of immunofluorescence staining in Fig 2A, increased infiltration of CD11b positive cells and NIMP-14 positive cells was observed the aorta and heart of 17 week old SOD3_R213G_ Tg mice, indicating that SOD3_R213G_ Tg mice have increased innate immune response with dominant infiltration of neutrophils. A similar pattern was observed in periphery (Fig 2B). Consistently, the expression of the chemo-attractants MCP-1, MIP1α, and MIP1β was up-regulated in the aorta and heart of the SOD3_R213G_ Tg mice (Fig 2C–2E). Correspondingly, the expression of inflammatory genes TNFα, IL-1β, and IL-6 was also up-regulated (Fig 2F–2H). In addition, the expression of elastase and the aging marker TERT was upregulated in the aorta and heart of SOD3_R213G_ Tg mice (Fig 2I and 2J), implying that 17 week old SOD3_R213G_ Tg mice exhibit a premature aging phenotype.

### Premature aging SOD3_R213G_ Tg mice develop neutrophilia

**Fig 3:**
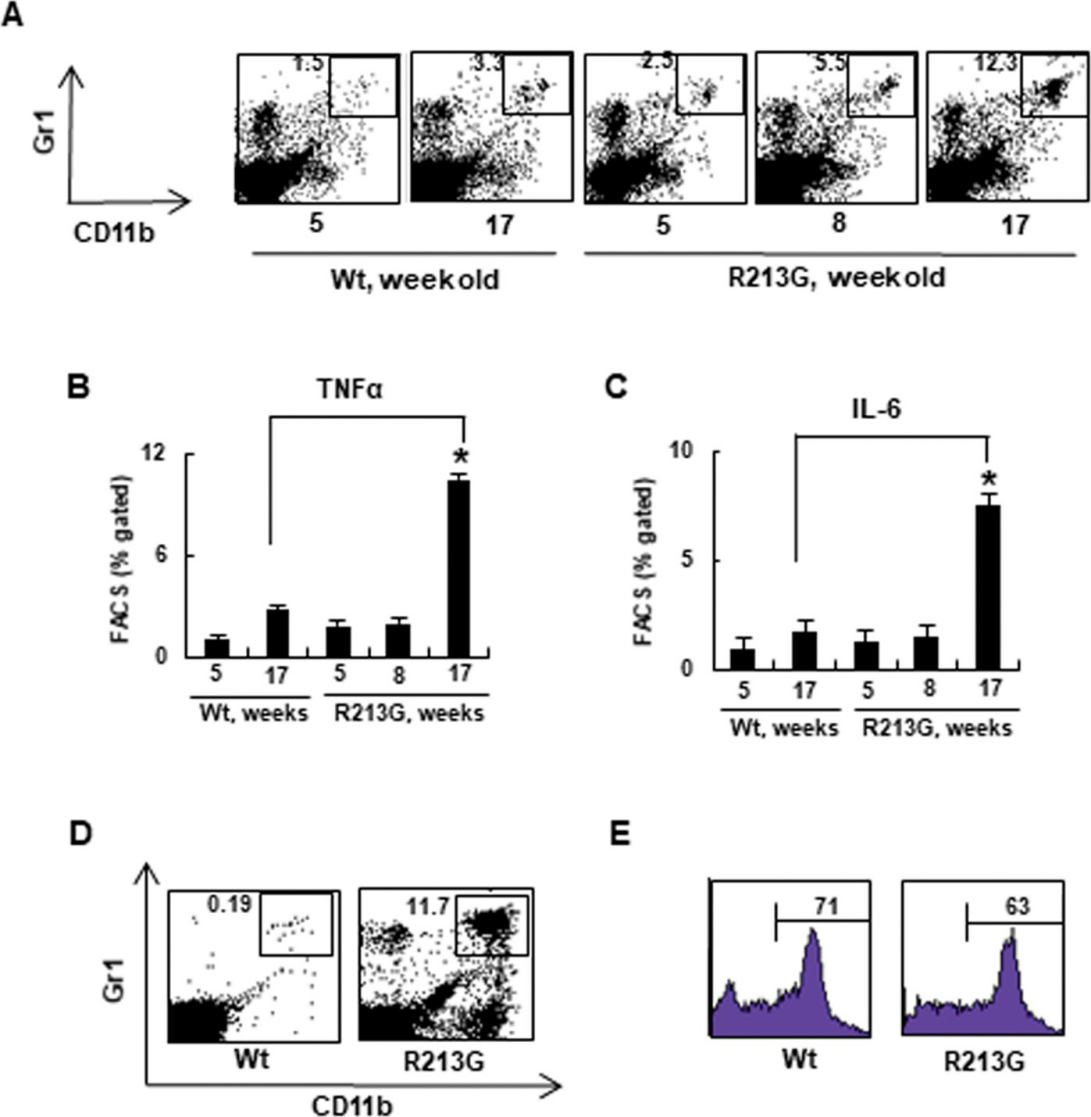
Premature aging SOD3_R213G_ mice develop neutrophilia. **A**. Changes in neutrophils levels of SOD3_R213G_ mice upon aging. Neutrophils (Gr1^+^CD11b^+^) in the spleen of SOD3_R213G_ and Wt mice were assessed by FACS analysis as described in Materials and Methods. **B-C**. Changes in proinflammatory cytokine levels of SOD3_R213G_ mice upon aging. The levels of cytokines, TNFα (B) and IL-6 (C) of SOD3_R213G_ and Wt mice at the indicated time were assessed by flow cytometry as described in Materials and Methods. **D-E**. Neutrophil content of SOD3_R213G._ mice. Neutrophils (Gr1^+^CD11b^+^) in the blood (D), and Gr+ bone marrow neutrophils (E) of 17 week old SOD3_R213G_ and Wt mice were assessed by FACS analysis as described in Materials and Methods. FACS data are representative of at least three independent experiments. Statistical analysis was performed by *t*-test (*p<0.001).

Consistently, increased numbers of Gr1^+^ CD11b^+^ neutrophils were observed in the spleen of the SOD3_R213G_ mice (Fig 3A). Correspondingly, the pro-inflammatory cytokines TNFα (Fig 3B) and IL-6 (Fig 3C) levels were increased. A similar pattern was observed in the periphery of SOD3_R213G_ mice (Fig 3D), but BM neutrophil content was not affected (Fig 3E). Thus, these results indicate that SOD3_R213G_ Tg mice may be sensitized by infection and have the potential for neutrophil-mediated inflammation. This implies that SOD3 may have a role in protecting against infection, and arginine at amino acid 213 in the HBD plays a key role in this function.

### SOD3_R213G_ alters SOD3 mediated signaling, which changes the function of neutrophils

Based on these results, we further investigated the role of SOD3_R213G_ at the molecular level. We hypothesized that SOD3_R213G_ may alter SOD3 mediated signaling pathways in neutrophils by changing SOD3 interacting proteins, which may, in turn, change their characteristics and functions. To test this possibility, neutrophils were isolated from 17 week old SOD3_R213G_ Tg or Wt mice and treated with G-CSF. The cells were lysed to perform immunoprecipitation using anti-SOD3 and bands were isolated for mass spectrometric analysis (S1 Fig). As we expected, SOD3_R213G_ expressing neutrophils had an altered profile of SOD3 interacting proteins in response to G-CSF treatment (S1 Table). Specifically, SOD3 interacted with membrane receptors and transporters, including T-complex protein, Arhgap21 protein, and synemin isoform HF (S1 Table), but these interactions were amplified in SOD3_R213G_ expressing neutrophils. In addition, SOD3_R213G_ interacted with the Usherin precursor, sodium bicarbonate cotransporter, docking protein 1, IRK2 channel protein, serine/threonine protein kinases TAO2 isoform 1, and G protein-coupled receptor 110, which did not interact with SOD3 (S1 Table). Thus, we further clarified these results at the cellular level.

**Fig 4:**
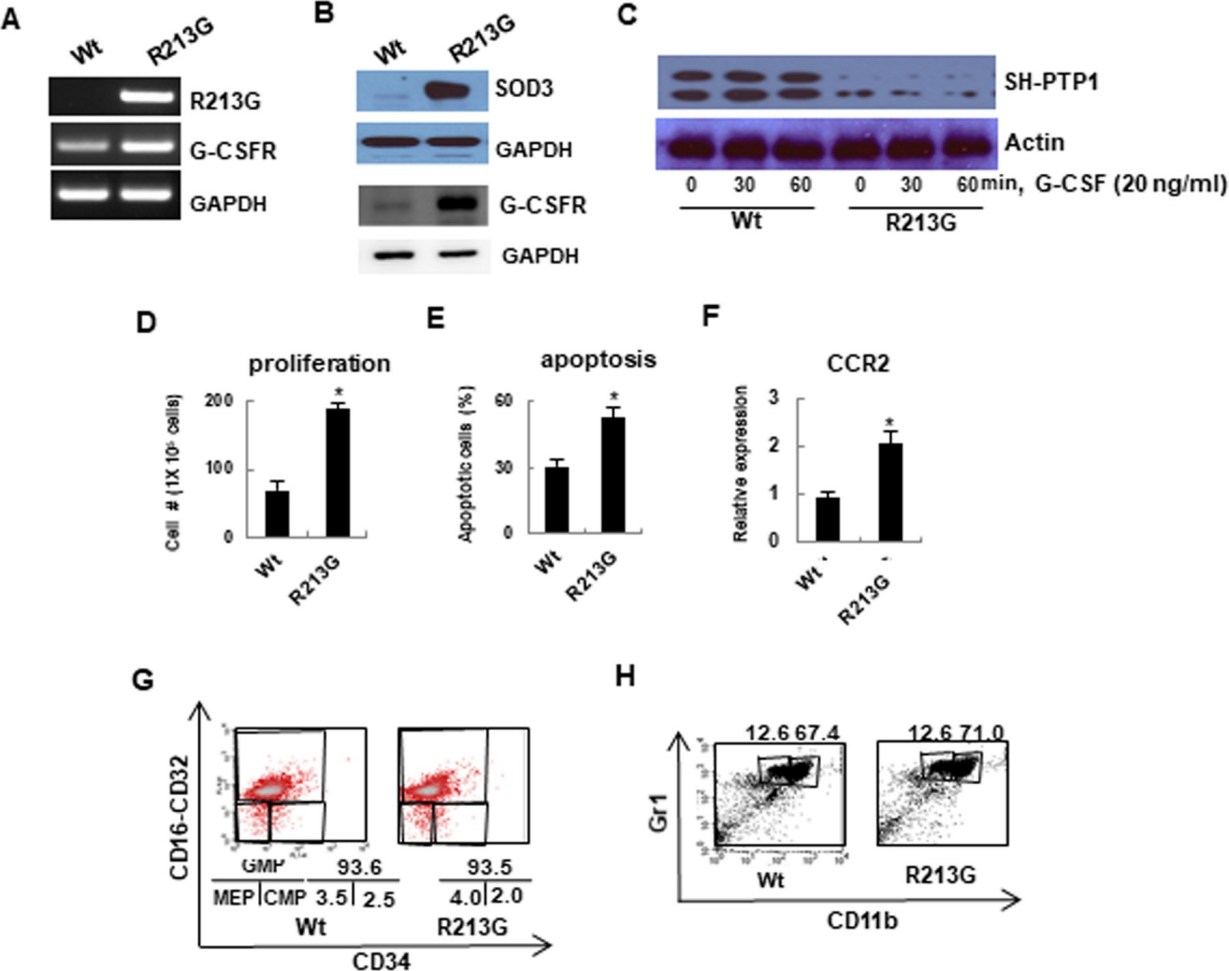
SOD3_R213G_ expressing neutrophils exhibit altered G-CSF mediated signaling, promoting proliferation, apoptosis, and trafficking. **A and B**. The expression of SOD3_R213G_ and G-CSFR in neutrophils. The expression of the indicated genes was assessed by RT-PCR (A) and immune blot (B) as described in Materials and Methods. **C**. Down regulation of SH-PTP1 signaling in neutrophils of 17 week old SOD3_R213G_ mice. G-CSF mediated Src tyrosine phosphatase, SH-PTP1 level (C, Top panel) was assessed by treating the neutrophils isolated from 17 week old SOD3_R213G_ or Wt mice with G-CSF (20 ng/ml) at the indicated time. The cells were lysed and subjected to SDS-PAGE, followed by immune blot with an antibody against SH-PTP1. **D-F**. Proliferation, apoptosis, and chemokine receptor CCR2 expression in neutrophils. The proliferation (D) and apoptosis (E) of neutrophils were measured by treatment with G-CSF (100 ng/ml) for 5 days as described in Materials and Methods. Chemokine receptor CCR2 expression (F) in neutrophils was assessed by qRT-PCR. **G** and **H**. Granulopoiesis and maturation of neutrophils were not affected by SOD3_R213G_. To assess granulopoiesis (G), BM isolated neutrophils from SOD3_R213G_ or Wt mice were subjected to FACS analysis with the indicated antibodies and myeloid progenitor cells were assessed. GMP: granulocyte-monocyte progenitors, CMP: common myeloid precursors, MEP: megakaryocyte-erythrocyte precursors. For the maturation analysis (H), CD11b^low^Gr1^high^ cells for immature and CD11b^high^Gr1^high^ cells for mature neutrophils were assessed by FACS analysis. RT-PCR, immunoblot, and FACS data are representative of at least three independent experiments. All qRT-PCR data were analyzed as described in Fig 1I–1M. Statistical analysis was performed by *t*- test (*p<0.001).

G-CSF and G-CSFR are critical in response of neutrophils to bacterial infection, controlling granulopoiesis and trafficking [26, 27]. Interestingly, G-CSFR was over-expressed in neutrophils isolated from 17 week old SOD3_R213G_ Tg mice compared to those of Wt mice (Fig 4A, middle panel). A consistent pattern was observed in the protein level (Fig 4B. middle panel). Surprisingly, G-CSF mediated Src homology protein tyrosine phosphatase, SH-PTP1, was down-regulated in SOD3_R213G_ expressing neutrophils (Fig 4C, top panel). Subsequently, the neutrophils showed increased proliferation (Fig 4D), apoptosis (Fig 4E), and expression of chemokine receptor CCR2 (Fig 4F), upregulating their trafficking. However, interestingly, SOD3_R213G_ did not affect granulopoiesis (Fig 4G) or maturation of neutrophils (Fig 4H). Taken together, these results suggest that SOD3_R213G_ expressing neutrophils have altered SOD3 mediated signaling in response to environmental pathogens, promoting proliferation and trafficking of neutrophils. Therefore, these results imply that SOD3 acts as a signaling molecule to control innate immune response, and arginine at amino acid 213 of HBD is critical for this function.

### Aberrant phenotype of SOD3_R213G_ Tg mice is recovered by reconstitution with SOD3 expressing BM cells

**Fig 5:**
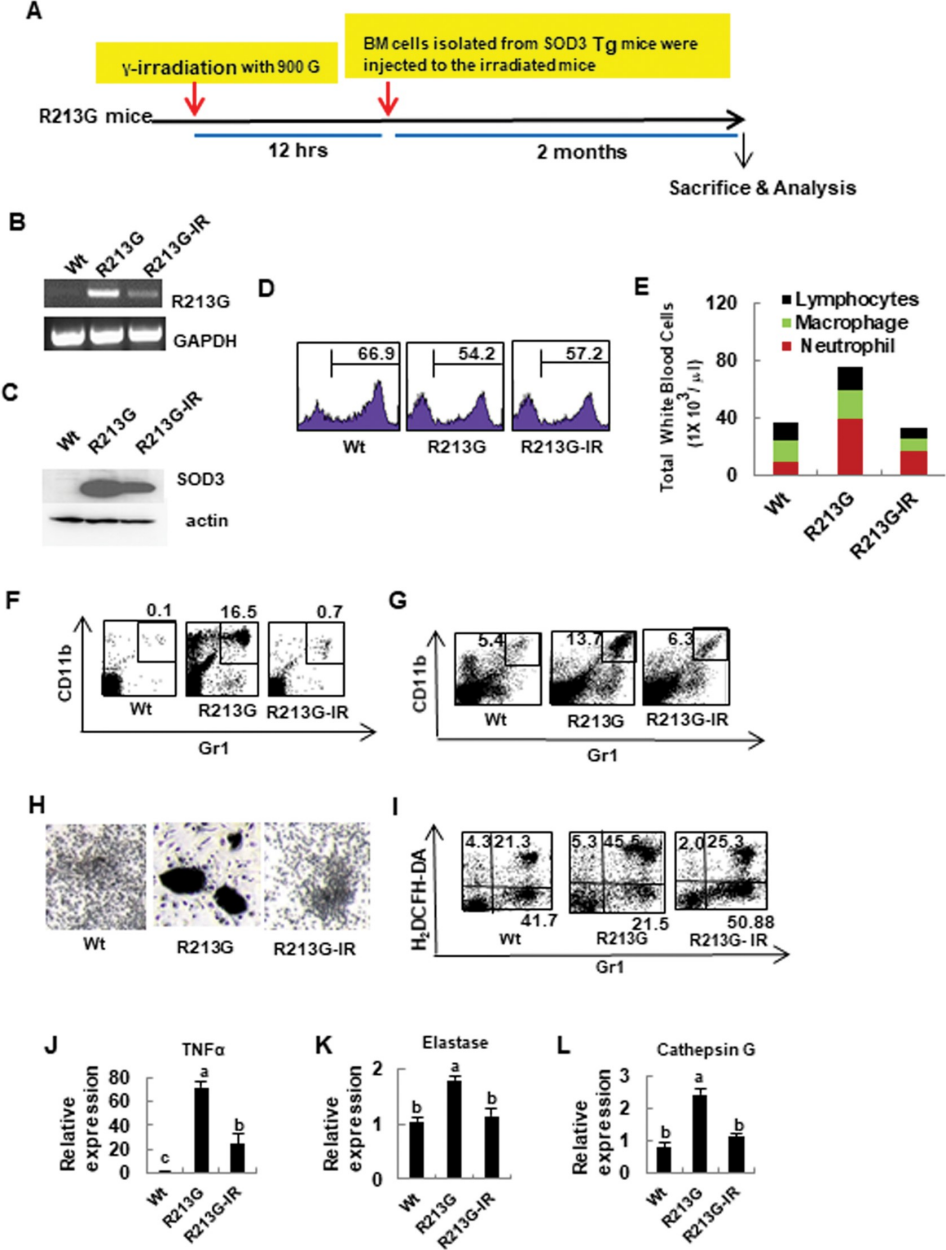
Aberrant neutrophils function of SOD3_R213G_ mice is recovered by reconstitution with SOD3 expressing BM cells. **A**. The scheme of BM reconstitution. Reconstitution of SOD3 expressing BM cells in SOD3_R213G_ mice was described in Materials and Methods. **B**. The expression of SOD3_R213G_. SOD3_R213G_ expression in neutrophils was assessed by RT-PCR as in Fig 4A. **C**. The protein expression of SOD3. The SOD3 protein expression in the BM neutrophils was assessed as described in materials and methods. **D**. The level of BM cells in transplanted SOD3_R213G_ mice. Gr1+ BM cells were assessed as described in Materials and Methods. **E**. Cell profiles in the blood. The cell profiles, including lymphocytes, macrophages, and neutrophils are assessed as described in Materials and Methods. **F** and **G**. The contents of neutrophils in the peripheral blood and spleen. Gr1^+^CD11b^+^ neutrophils in the blood (F) and spleen (G) were assessed by FACS analysis as described in Fig 3A and 3D. **H**. Morphological changes of neutrophils. BM cells were isolated from 17 week old SOD3_R213G_, transplanted, and Wt mice, and treated with G-CSF as described in Materials and Methods. The morphological image was taken by light microscopy. Scale bar, 200 μm. **I.** Changes in ROS level. ROS generation was assessed by treatment with fMLP (100 nM, 1 hr) in the BM cells, and stained with H_2_DCFH-DA, 2’,7’-dichlorofluorescin diacetate (5 μM) with Gr1 and analyzed by FACS as described in Materials and Methods. **J-L.** Gene expressions of neutrophils in SOD3_R213G_ mice. The expression of TNFα (J), elastase (K), and cathepsin G (L) in neutrophils was measured by qRT PCR. RT-PCR and FACS data are representative of at least three independent experiments. All qRT PCR data were analyzed as in Fig 1I–1M. Statistical analysis was performed by using ANOVA at *p*<0.05, followed by Scheffe’s post hoc test. R213G-IR represents transplanted SOD3_R213G_ mice.

A study showed that SOD3 gene transfer to damaged tissue results in increased healing [28]. In addition, treatment of cardiovascular tissues with SOD3 reduces the extent of the damage, increases the healing process, and improves cardiac function [28]. Based on that information and to confirm the pathologic properties of SOD3_R213G_ and its molecular mechanisms, BM stem cells were isolated from 17 week old SOD3 Tg mice and transplanted into lethally irradiated same background and same age SOD3_R213G_ Tg recipient mice (Fig 5A). Two months later, SOD3_R213G_ expression was assessed in transplanted mice. As shown in Fig 5B and Fig 5C, SOD3_R213G_ expression was drastically decreased in BM transplanted SOD3_R213G_ mice. Using FACS analysis, we found that the transplanted mice have a similar level of BM cells to SOD3_R213G_ mice (Fig 5D), indicating that transplanted BM cells restored normal granulopoiesis in γ-irradiated SOD3_R213G_ Tg mice. The profile of blood immune cells of transplanted mice was similar to Wt (Fig 5E). As we expected, the numbers of neutrophils in the periphery (Fig 5F) and spleen (Fig 5G) were dramatically reduced in the transplanted mice compared to SOD3_R213G_ Tg mice. Consistently, activated blast-like form and highly aggregated neutrophils in SOD3_R213G_ reverted to the Wt phenotype (Fig 5H), and increased ROS level in neutrophils of SOD3_R213G_ Tg mice returned to the Wt levels (Fig 5I). Subsequently, the expression level of pro-inflammatory and tissue damaging molecules TNFα (Fig 5J), elastase (Fig 5K), and cathepsin G (Fig 5L) in neutrophils of SOD3_R213G_ Tg mice, was drastically reduced by transplantation.

### Altered signaling and aberrant function of the neutrophils are recovered by reconstitution with SOD3 expressing BM cells, which ultimately recover the cardiovascular system

**Fig 6:**
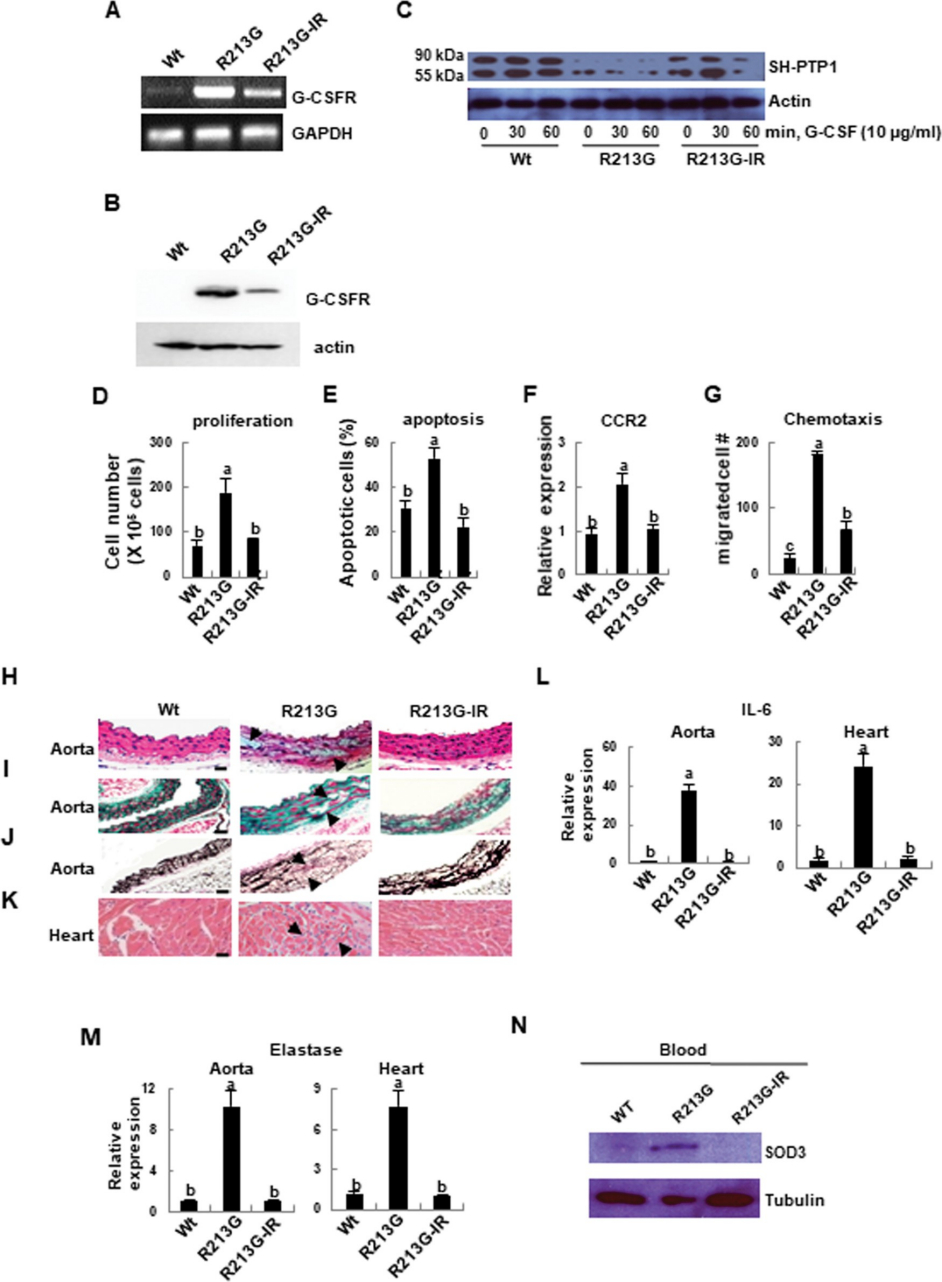
Altered signaling of neutrophils in SOD3_R213G_ mice is recovered by reconstitution with SOD3 expressing BM cells, and aortic degeneration and heart inflammation are recovered. **A** and **B**. G-CSFR expression of neutrophils in SOD3_R213G_ mice was drastically reduced by BM transplantation. G-CSFR gene expression (A) and protein level (B) of neutrophils in Wt, SOD3_R213G_, and SOD3_R213G_-IR mice was assessed as described in Fig 4A. **C**. Altered G-CSF mediated SH-PTP1 signaling of neutrophils of SOD3_R213G_ mice was recovered by BM transplantation. G-CSF mediated SH-PTP1 levels (top panel) in neutrophils of Wt, SOD3_R213G_, and SOD3_R213G_-IR mice, were assessed as described in Fig 4C. **D-G**. Neutrophils function was recovered by BM transplantation. Proliferation (D), apoptosis (**E**), and chemokine receptor CCR2, expression (**F**) in neutrophils were measured and analyzed as described in Fig 4D–4F. Chemotaxis (G) of neutrophils was assessed as described in Materials and Methods. **H-K**. Aortic degeneration and heart inflammation were recovered by transplantation. Abdominal aorta (H-J) and heart (K) of Wt, SOD3_R213G_, and R213G-IR mice were isolated. H & E (H and K), Masson’s trichrome (I), and Van Grieson staining (J) were performed as described in Fig 1A–1D. Scale bar, 50 μM. L and M. Gene expression of pro-inflammatory molecules. The expression of IL-6 (L) and elastase (M) in the aorta and heart were assessed by qRT-PCR as described in Fig 1I–1M. N. SOD3 level in the blood of SOD3_R213G_ mice was drastically reduced by BM transplantation. Blood isolated from Wt, SOD3_R213G_, or transplanted mice was lysed and SDS-PAGE was performed, followed immunoblot with indicated antibodies. All images are representative of at least three independent experiments. Each group has three to four Wt, SOD3_R213G_, or transplanted mice (R213G-IR) mice for an experiment. Statistical analysis was performed by using ANOVA at *p*<0.05, followed by Scheffe’s post hoc test, grouping a, b, and c.

As we expected, up-regulated G-CSFR expression in the neutrophils of SOD3_R213G_ Tg mice was drastically reduced by transplantation (Fig 6A and 6B, top panel). In addition, down-regulation of G-CSF mediated tyrosine phosphatase SH-PTP1 in neutrophils of SOD3_R213G_ Tg mice was recovered by reconstitution with SOD3 expressing BM cells (Fig 6C, top panel). Consistently, aberrant neutrophils function, including proliferation (Fig 6D), apoptosis (Fig 6E), CCR2 expression (Fig 6F), and chemotaxis (Fig 6G), returned to Wt level by transplantation.

Finally, vascular pathologic changes including aortic myocyte degeneration, cystic medial degeneration, and heart inflammation in SOD3_R213G_ Tg mice were recovered by transplantation (Fig 6H–6K). Correspondingly, increased levels of the pro-inflammatory cytokine IL-6 (Fig 6L) and elastase (Fig 6M) in the aorta and heart were also significantly reduced by transplantation. Moreover, SOD3 in the blood of SOD3_R213G_ Tg mice was reduced to Wt level by the transplantation (Fig 6N, top panel). Taken together, these results confirmed that the pathologic properties of SOD3_R213G_ and its molecular mechanism are recovered by transplantation of SOD3 expressing BM cells.

## Discussion

**Fig 7:**
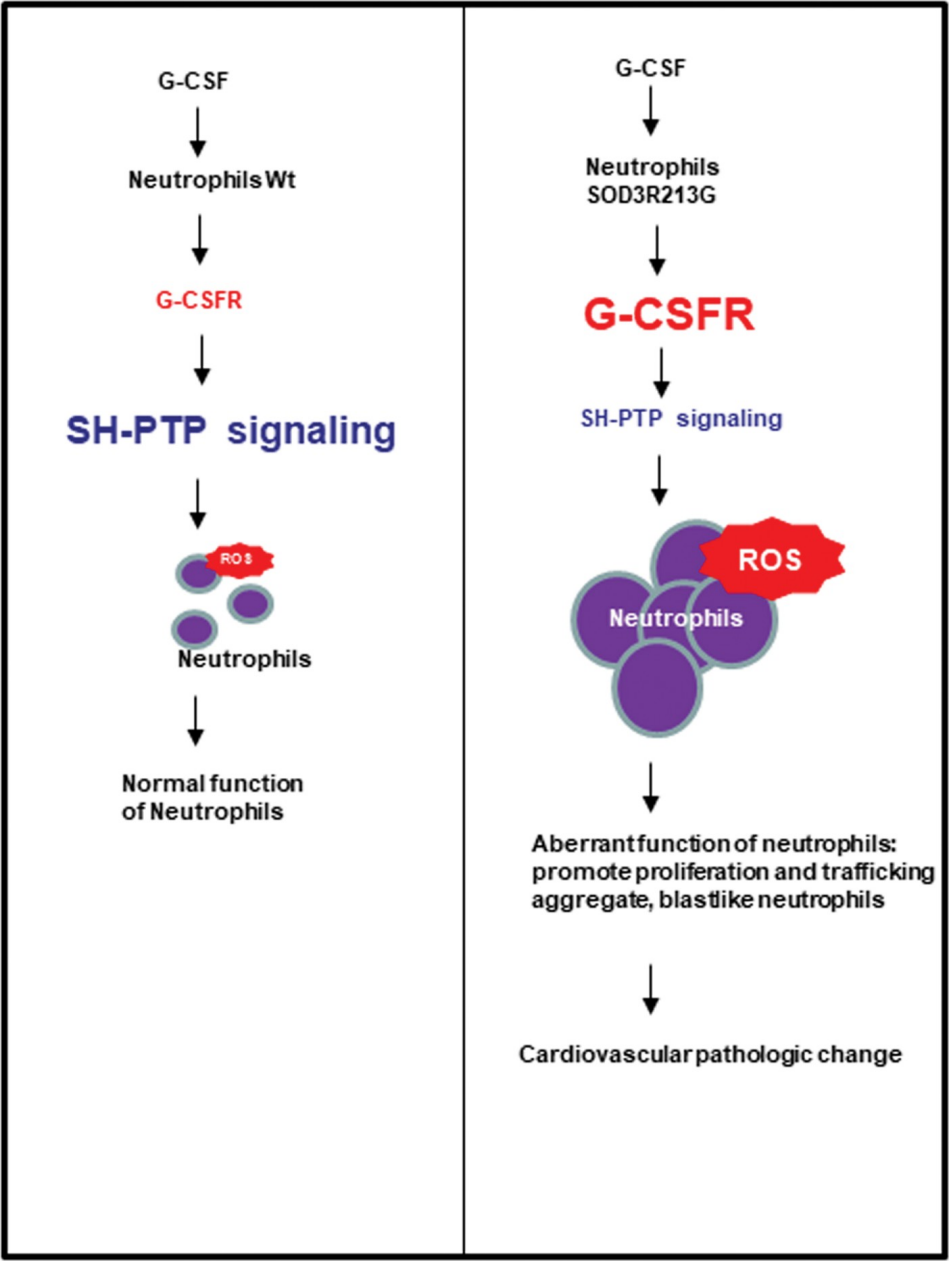
Proposed mechanism of cardiovascular pathologic change in SOD3_R213G_ mice. Neutrophils of SOD3_R213G_ mice are dominantly infiltrated in their organs in response to G-CSF, promoting proliferation and trafficking to their organs. Neutrophils of SOD3_R213G_ mice are sensitized, and down-regulated SH-PTP signaling in response to G-CSF may play a major role.

In the present study, we demonstrated that overexpression of SOD3_R213G_ causes aortic degeneration and heart inflammation, which eventually leads to loss in cardiovascular function. One of the mechanisms we proposed is that overexpression of SOD3_R213G_ causes increased proliferation and trafficking of neutrophils by altering protein interactions with SOD_R213G_ and down-regulating SH-PTP1 signaling (**Fig 7**). These effects were recovered by bone marrow transplantation (BMT) with Wt SOD3.

A key feature of SOD3 protein structure is the carboxy-terminus containing 6 positively charged amino acids, which is essential for anchoring to the negatively charged ECM [29]. SOD3_R213G_ disrupts the positive charge of SOD3 and reduces the affinity for the ECM, leading to its redistribution to the extracellular fluid space, such as plasma and epithelial lining fluid [29]. In agreement with our finding, it was reported that SOD3 reduces the superoxide level and improves aortic relaxation in response to LPS treatment, but SOD3_R213G_ fails to protect against endothelial dysfunction [30]. As a possible mechanism, it has been reported that SOD3_R213G_ alters the affinity of the HBD for heparin via conformational change [31]. A study showed that SOD3_R213G_ binds to bovine aortic endothelial cells 50 fold less than SOD3 [32]. In addition, SOD3 is released into the blood in humans who carry the SOD3_R213G_ variant [14, 32, 33], which is consistent with our result. However, it has also been reported that SOD3_R213G_ does not change SOD3 functions, but only in its distribution [29], and SOD3_R213G_ attenuates the risk of exacerbation of COPD [29] and protects against allergic airway inflammation [29]. This discrepancy may be due to different organs, different morbidity, and redistribution of SOD3 into extracellular fluids which play a critical role in the function of the lung. Thus, it seems that proper affinity of SOD3 to the ECM is essential for the prevention of inflammatory and degenerative diseases in the cardiovascular system.

Unlike Wt SOD3, we observed that SOD3_R213G_ Tg mice promotes innate immune responses. Specifically, higher numbers of neutrophils infiltrated to the organs of SOD3_R213G_ Tg mice over time, which promotes ROS generation and secretion of pro-inflammatory and tissue damaging molecules. Consistently, a study has shown that SOD3 inhibits innate immune response by decreasing colocalizing of neutrophils with bacteria and increasing neutrophil apoptosis, reducing neutrophil-specific TNFα and peroxynitrite production [34]. Considering that neutrophils are involved in many parts of cardiovascular pathophysiology [35] and elevated blood neutrophils are a predictor of cardiovascular death in patients with coronary artery disease [35], it seems that the fundamental function of SOD3 under physiological conditions is controlling immune response against microbial invasion. However, the present study showed that SOD3_R213G_ Tg mice does not affect neutrophil development. Instead, it affects proliferation and trafficking of neutrophils. A supportive study suggested that SOD3 mRNA is absent throughout neutrophil maturation, but it is synthesized in other cells and subsequently endocytosed by the neutrophils [36]. Thus, further investigation of role of SOD3 in immune cells is warranted.

In this study, all mice were maintained in a semi-specific pathogen-free (SPF) condition. However, it is likely that SOD3_R213G_ Tg mice still experienced immune sensitizing in that environment, which initiates up-regulation of G-CSF mediated signaling and alters interacting proteins. As a consequence, SOD3_R213G_ expressing neutrophils changed their functions, leading to a progressive deterioration of the aorta and heart. In addition, higher neutrophils levels in the periphery resulted in increased blood levels of SOD3. Supporting our findings, G-CSF has been shown to promote mobilization of neutrophils from the BM to the blood [37]. Moreover, many signaling molecules, including JAK, Src family tyrosine kinases, PI3/Akt, and Ras/MAPK pathways participate in the G-CSFR mediated signal transduction cascade [38, 39]. Furthermore, IL-1β-Myd 88 axis and NADPH oxidase-mediated ROS signaling regulate neutrophil migration [40]. Thus, further investigation is warranted to clarify the immune cell profile of SOD3_R213G_ Tg mice and demonstrate how SOD3_R213G_ altered signaling pathways against pathogens.

However, while Tg mice which express SOD3 lacking HBD have a similar phenotype to SOD3 Tg mice, a functional difference of SOD3 was observed in SOD3_R213G_ Tg mice [25]. Although SOD3 Knock out (Ko) mice were susceptible to inflammation, they do not present similar phenotypes to SOD3_R213G_ Tg mice, which may be due to the functional redundancy of SOD3. In other words, other SODs, such as SOD1 or SOD2, may compensate to promote protective effects in both SOD3 lacking HBD Tg and SOD3 Ko mice. Indeed, SOD1 and SOD3 are both belong to Cu, Zn- SOD and have 50% homologues each other [41]. In addition, study showed that while SOD2 Ko (SOD2^-/-^) mice are lethal within 3–4 week after birth, SOD1 Ko or SOD3 Ko mice are not lethal [42]. This suggests that compensatory mechanism is operating at least for SOD1 and SOD3. Furthermore, we observed the unique phenotype and altered the neutrophils function in SOD_R213G_ overexpressing mice whose generated by universal promoter, instead of generating target specific knock-in mice. Thus, we may further clarify if there is discrepancy between these mice. Taken together, these findings suggest that SOD3 may play a protective role by controlling immune response, at least in part, through orchestration of proper signaling in response to environmental pathogens. In that sense, arginine 213 in the HBD of SOD3 is essential for the signaling function of SOD3.

We demonstrated that SOD3_R213G_ mediated aortic degeneration can be recovered by reconstitution with SOD3 expressing BM cells. A supportive study reported that SOD3 gene transfer using an adenoviral vector reduces the extent of cardiovascular damage, improves cardiac function, reducing remodeling of vasculature, attenuating apoptosis, inhibiting inflammatory and smooth muscle cell migration, and increasing cell proliferation and endothelial cell layer recovery [28]. Regarding this, it has been shown that although the remaining myocytes are unable to repair necrotic tissue, the injured organ is sensed by distant stem cells, which migrate to the site of damage and undergo alternative stem cell differentiation [43, 44]. Supportive studies showed that injection of Lin^-^c-kit^pos^ BM cells in infarcted mice regenerate the myocardium [45]. However, gene therapy has limitations as an approach due to its safety and delivery issues [46], which led us to apply bone marrow transplantation.

In summary, the present study shows that SOD3_R213G_ Tg mice has pathogenic characteristics in the cardiovascular system by increasing sensing infection, increasing proliferation and trafficking of neutrophils and altering their signaling pathways. Therefore, arginine 213 of HBD of SOD3 is critical for the function of SOD3 in the cardiovascular system, and SOD3 expressing BMT may be a potential therapeutic strategy.

## Supporting information

S1 Fig Image of SDS-PAGE gel to analyze through mass spectrometry. **A**. Neutrophils were isolated from BM of 17 week old Wt or SOD3_R213G_ mice and treated with G-CSF (100 ng/ ml) for 30 min. SOD3 interacting proteins were pulled down with anti-SOD3 followed by SDS PAGE as described in Materials and Methods. To detect SOD3 interacting signaling molecules, bands around 35–47 kDa and 75–95 kDa were excised and analyzed by mass spectrometry.
(TIF)

S2 Fig SOD activity in the aorta, heart, and blood. **A-B**. SOD activity in the aorta and heart (A) and blood (B) in Wt, SOD3_R213G_, and R213G-IR mice. SOD activity in the tissue or blood was measured as described in the Materials and Methods. **C**.SOD3, SOD2, SOD1, and nitrotyrosine expression of aorta and heart of Wt, SOD3_R213G_, or R213G-IR mice. The level of SODs and nitrotyrosine in the aorta and heart of Wt, SOD3_R213G_, or R213G-IR mice was assessed by SDS-PAGE and immunoblot with indicated antibodies. The membrane was reprobed and immunoblot against Tubulin was performed.
(TIF)

S3 Fig FACS analysis showing that BMT mice did not exhibit allograft reaction. **A**. MHC II and MHC I (H2-Kb) expression in dendritic cells of SOD3 Tg and SOD3_R213G_ mice. **B**. Splenic CD8 and B220 expression of SOD3_R213G_ mice and R213G-IR mice. **C-E**. Splenic proinflammatory cytokine profiles, IL-4 and IL-13 (C), IL-17 and IL-6 (D), and TNFα (E) of SOD3_R213G_ mice and R213G-IR mice. R213G-1, R213G-2, and R213G-3 represent individual SOD3_R213G_ mice. R213G-IR1, R213G-IR2, and R213G-IR3 represent individual bone marrow transplanted mice.
(TIF)

S1 Table List of proteins that interact with SOD3_R213G_, compared with those of SOD3 in neutrophils in response to G-CSF. (DOC)
