# Supplementary figures and images for: Pathologic properties of SOD3 variant R213G in the cardiovascular system through the altered neutrophils function
Source: PLoS One. 2020 Jan 31;15(1):e0227449. doi: 10.1371/journal.pone.0227449 (PMC6994104; doi:10.1371/journal.pone.0227449)

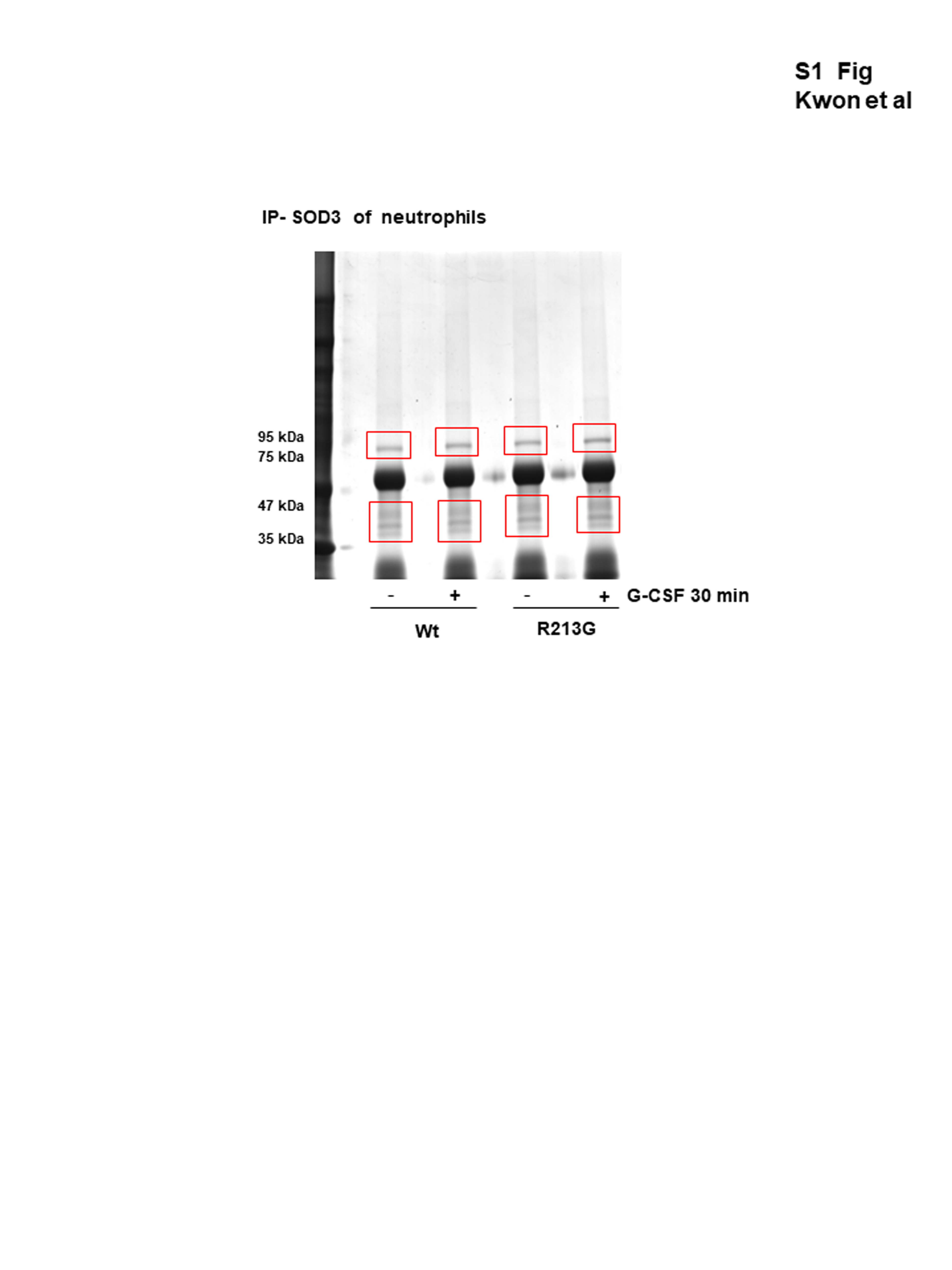

Supplement: S1 Fig — A. Neutrophils were isolated from BM of 17 week old Wt or SOD3R213G mice and treated with G-CSF (100 ng/ ml) for 30 min. SOD3 interacting proteins were pulled down with anti-SOD3 followed by SDS PAGE as described in Materials and Methods. To detect SOD3 interacting signaling molecules, bands around 35–47 kDa and 75–95 kDa were excised and analyzed by mass spectrometry. (TIF) [file pone.0227449.s001.tif]

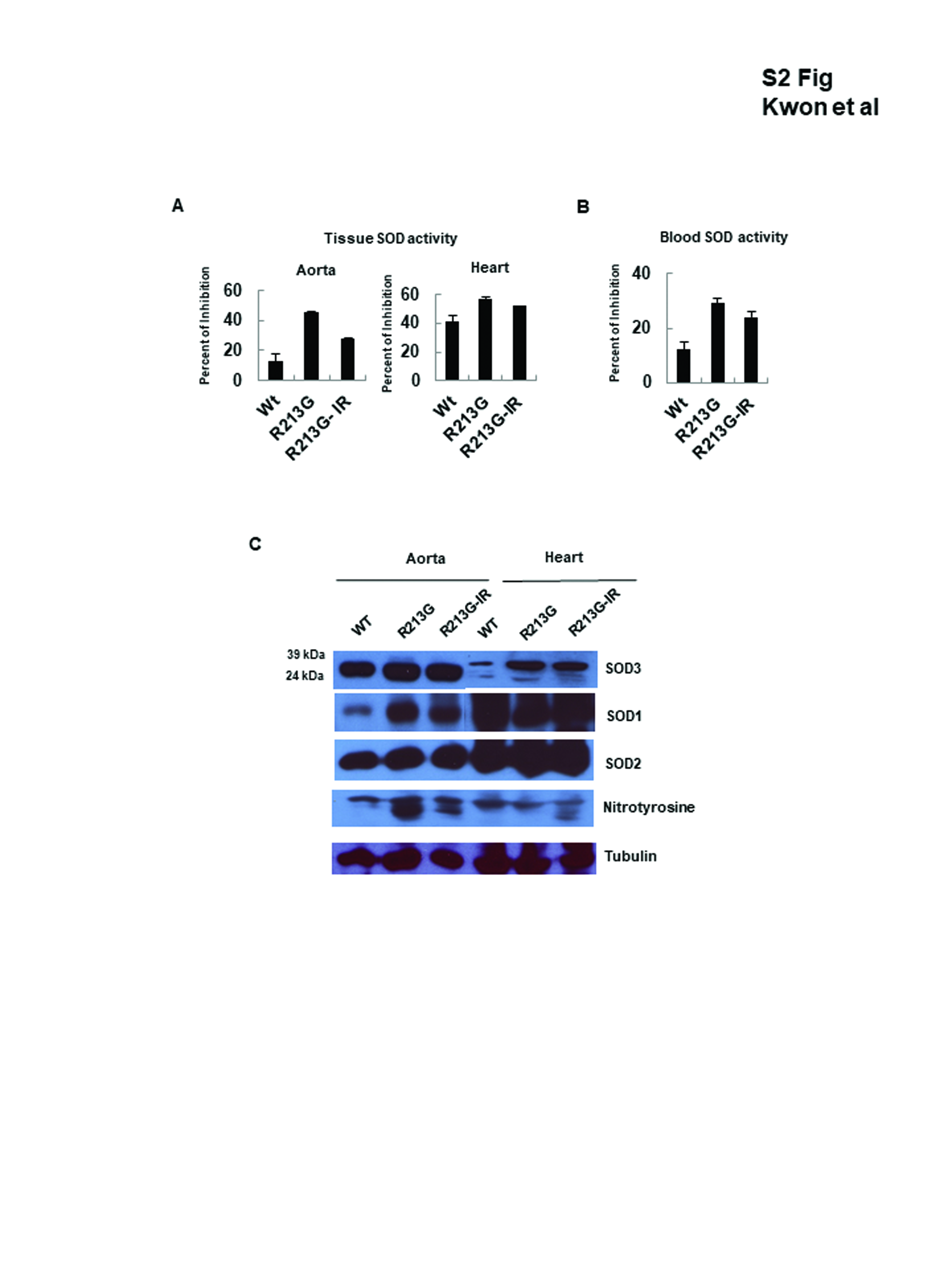

Supplement: S2 Fig — A-B. SOD activity in the aorta and heart (A) and blood (B) in Wt, SOD3R213G, and R213G-IR mice. SOD activity in the tissue or blood was measured as described in the Materials and Methods. C.SOD3, SOD2, SOD1, and nitrotyrosine expression of aorta and heart of Wt, SOD3R213G, or R213G-IR mice. The level of SODs and nitrotyrosine in the aorta and heart of Wt, SOD3R213G, or R213G-IR mice was assessed by SDS-PAGE and immunoblot with indicated antibodies. The membrane was reprobed and immunoblot against Tubulin was performed. (TIF) [file pone.0227449.s002.tif]

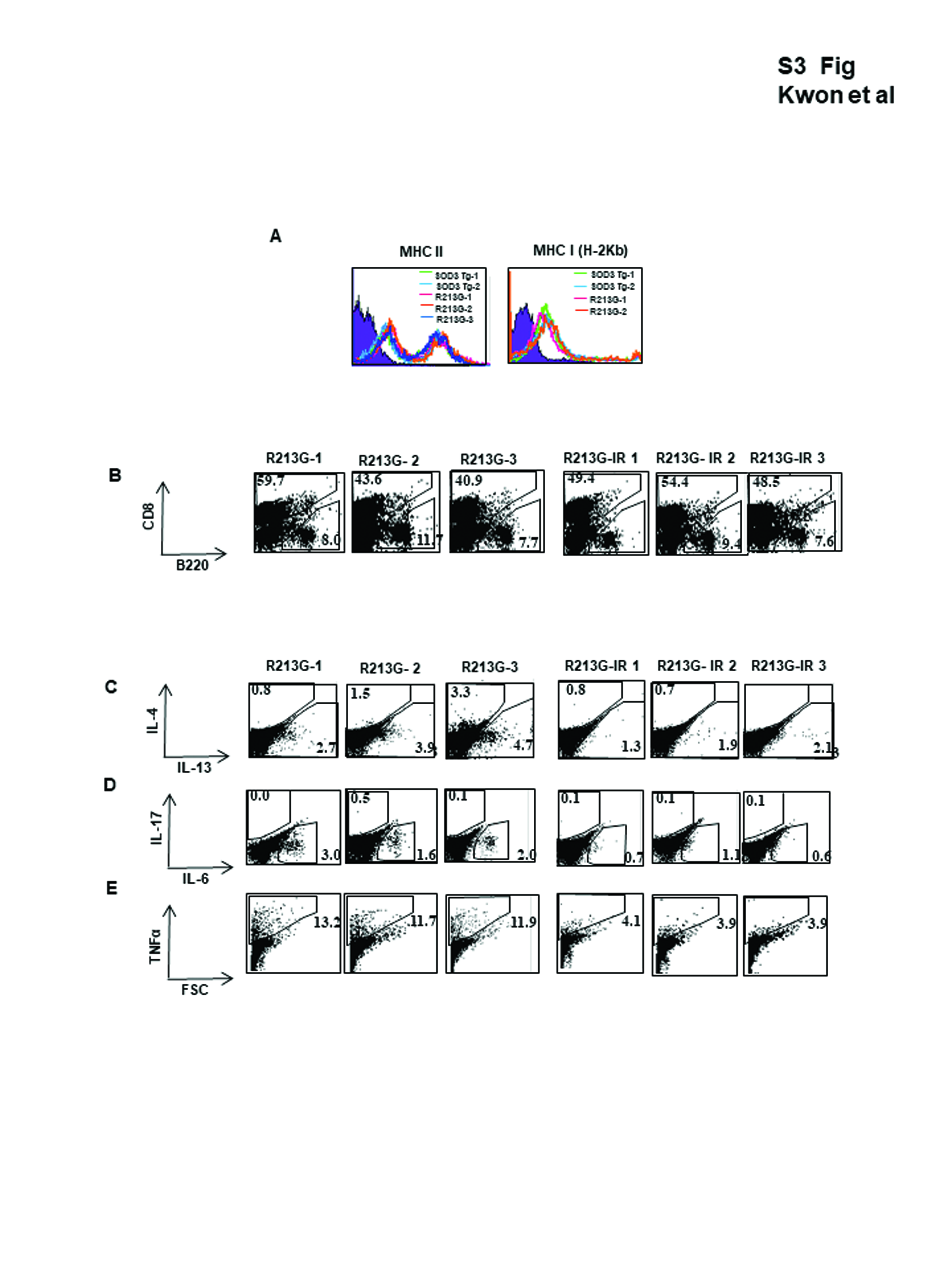

Supplement: S3 Fig — A. MHC II and MHC I (H2-Kb) expression in dendritic cells of SOD3 Tg and SOD3R213G mice. B. Splenic CD8 and B220 expression of SOD3R213G mice and R213G-IR mice. C-E. Splenic proinflammatory cytokine profiles, IL-4 and IL-13 (C), IL-17 and IL-6 (D), and TNFα (E) of SOD3R213G mice and R213G-IR mice. R213G-1, R213G-2, and R213G-3 represent individual SOD3R213G mice. R213G-IR1, R213G-IR2, and R213G-IR3 represent individual bone marrow transplanted mice. (TIF) [file pone.0227449.s003.tif]
