# Supplementary material for: Pathologic properties of SOD3 variant R213G in the cardiovascular system through the altered neutrophils function
Source: PLoS One. 2020 Jan 31;15(1):e0227449. doi: 10.1371/journal.pone.0227449 (PMC6994104; doi:10.1371/journal.pone.0227449)
Supplement: S1 Table — (DOC) [file pone.0227449.s004.doc]

**S1 Table. List of proteins that interact with SOD3_R213G_, compared with those of SOD3 in neutrophils in response to G-CSF**

| ***Identified proteins*** | ***Accession number*** | ***Mw (kDa)*** | ***Wt^a^*** | | ***SOD3_R213G_^b^*** | |
| --- | --- | --- | --- | --- | --- | --- |
|  |  |  | **-** | **+** | **-** | **+** |
| t-complex protein 10b | gi|130497908 | 49 | 0 | 1.64 | 1.25 | 3.08 |
| transient receptor potential cation channel, subfamily M, member 6 | gi|148709622 | 228 | 0 | 0 | 4.99 | 2.3 |
| Usherin precursor | gi145699097 | 570 | 0 | 0 | 0 | 7.28 |
| AP-4 complex subunit epsilon-1 | gi|124487335 | 125 | 0 | 0 | 0 | 1.54 |
| sodium bicarbonate cotransporter NBC1 | gi|5748813 | 116 | 1.28 | 0 | 1.25 | 2.31 |
| Arhgap21 protein | gi|187956405 | 217 | 0 | 1.64 | 0 | 2.31 |
| transcription factor C1 (HCF) | gi|1293686 | 211 | 0 | 0 | 0 | 3.1 |
| Olfactory receptor 315 | gi|111600698 | 34 | 1.28 | 0 | 1.25 | 0.77 |
| synemin isoform H | gi|41687953 | 173 | 0 | 1.64 | 0 | 0.77 |
| SAPS domain family, member2, isoform CRA | Gi16630486 | 52 | 0 | 0 | 2.49 | 0.77 |
| docking protein 1 | gi|148666630 | 52 | 0 | 0 | 2.49 | 0.77 |
| mCG64727, isoform CRA_b | gi|148688607 | 94 | 0 | 0 | 2.49 | 0.77 |
| RecName: Full=ATP-binding cassette sub-family A member 8-B | gi|115503761 | 183 | 0 | 0 | 0 | 1.53 |
| serine/threonine-protein kinase TAO2 isoform 1 | gi|255003682 | 120 | 0 | 0 | 0 | 2.31 |
| Sept12 protein | gi|109731886 | 19 | 0 | 0 | 0 | 2.31 |
| IRK2 channel | gi|6624203 | 47 | 0 | 0 | 1.25 | 0.77 |
| galanin receptor 1, isoform CRA_a | gi|148677441 | 39 | 0 | 0 | 0.77 | 0.31 |
| calsarcin-3 | gi|18767240 | 27 | 0 | 0 | 0 | 1.82 |
| copine V | gi|148690664 | 66 | 0 | 0 | 0 | 0.77 |
| centromere-associated protein E | gi|115648101 | 286 | 0 | 0 | 0 | 1.82 |
| Dkc1 protein | gi|71534070 | 57 | 0 | 0 | 0 | 1.82 |
| Cacna2d1 protein | gi|109732367 | 123 | 0 | 0 | 0 | 1.54 |
| tensin 1 | gi|226437589 | 201 | 0 | 0 | 0 | 1.54 |
| G protein-coupled receptor 110 | gi|148691453 | 99 | 0 | 0 | 0 | 1.54 |

^a, b^ Isolation of neutrophils from the Wt or SOD3_R213G_ mice and treatment with G-CSF as described in figure 1S. The number shown represents a quantitative value that was determined by normalizing spectra counts through Scaffold software analysis. Proteins were identified with 90% cut off range and unknown proteins were excluded. (- ) without or (+) with treatment of G-CSF
